# Cinnamon as a Complementary Therapeutic Approach for Dysglycemia and Dyslipidemia Control in Type 2 Diabetes Mellitus and Its Molecular Mechanism of Action: A Review

**DOI:** 10.3390/nu14132773

**Published:** 2022-07-05

**Authors:** Maria Leonor Silva, Maria Alexandra Bernardo, Jaipaul Singh, Maria Fernanda de Mesquita

**Affiliations:** 1 Centro de Investigação Interdisciplinar Egas Moniz, Instituto Universitário Egas Moniz, Campus Universitário, Quinta da Granja, Monte de Caparica, 2829-511 Caparica, Portugal; lsilva@egasmoniz.edu.pt (M.L.S.); abernardo@egasmoniz.edu.pt (M.A.B.); 2 School of Natural Sciences, University of Central Lancashire, Preston PR1 2HE, UK; jsingh3@uclan.ac.uk

**Keywords:** cinnamon, blood glucose, lipid profile, type 2 diabetes, anti-inflammatory, intervention studies

## Abstract

The scientific evidence that cinnamon may exert beneficial effects on type 2 diabetes mellitus due to the biological activity of its bioactive compounds has been increasing in recent years. This review provides an overview of the effects of cinnamon on clinical parameters of diabetes and summarizes the molecular mechanisms of action of cinnamon on glucose and lipid metabolism. Search criteria include an electronic search using PubMed, Medline, and Cochrane Library databases. English literature references from 2000 up to 2022 were included. Following title and abstract review, full articles that met the inclusion criteria were included. The results from the available evidence revealed that cinnamon improved glycemic and lipidemic indicators. Clinical trials clarified that cinnamon also possesses an anti-inflammatory effect, which may act beneficially in diabetes. Based on *in vitro* and *in vivo* studies, cinnamon seems to elicit the regulation of glucose metabolism in tissues by insulin-mimetic effect and enzyme activity improvement. Furthermore, cinnamon seems to decrease cholesterol and fatty acid absorption in the gut. The current literature search showed a considerable number of studies on diabetic subjects. Some limitations in comparing published data should be highlighted, including variability in doses, extracts and species of cinnamon, administration forms, and antidiabetic therapy.

## 1. Introduction

Type 2 diabetes mellitus (T2DM) is a major global health disorder, and it constitutes an important contribution to morbidity and mortality worldwide. T2DM is identified to be part one of the ten top diseases leading to disability, morbidity, and mortality (DMM) in all age groups. In 2017, death and disability-adjusted life years (DALY) related with diabetes were 1.37 million and 67.9 million, with a projection of 1.59 million and 79.3 million in 2025, respectively, in which inappropriate diet was one of the most contributing factors [1]. It is estimated that the global prevalence of T2DM estimates for adults between 20 and 79 years will increase worldwide from 480 million in 2022 to 692 million by 2035 [2].

Diabetes is a metabolic disorder resulting from defects in insulin secretion and/or action, leading to chronic hyperglycemia (HG), which has an adverse impact on health [3]. In this context, early detection and treatment are fundamental in management and, more so, in avoiding the progress of the disorder and in the development of long-term complications, which usually appear later in the disorder [4].

For centuries, traditional medicines have been efficiently used as an alternative and/or complement in T2DM treatment [5]. Bioactive compounds isolated from plants have been shown to exert antidiabetic activity due to their effect on lowering glucose levels [6]. One such plant is cinnamon, which is a spice that has been used in culinary in several cultures. In addition to its traditional cuisine uses, cinnamon has been shown to exert a potential effect on T2DM management [7,8]. Published data have shown that cinnamon possesses a hypoglycemic effect in T2DM [9]. However, cinnamon supplements demonstrated conflicting and contradicting results in diabetic patients [10]. A number of phytochemical compounds extracted from cinnamon have demonstrated potential antidiabetic properties. One such compound is cinnamaldehyde, which is a water-soluble polyphenol [11]. Polyphenolic compounds possess anti-inflammatory properties through regulation of interleukin 1 and 6 (IL-1 and 6), C-reactive protein (CRP), and tumor necrosis factor (TNF)-alpha level [12]. The beneficial properties of cinnamon polyphenols have also been attributed to their antioxidant proprieties [13,14]. 

This narrative review provides an overview of the beneficial effects of cinnamon in exerting dysglycemia and dyslipidemia control in type 2 diabetic patients and a summary of its mechanisms of action. 

## 2. Search Methodology

### 2.1. Study Design and Search Criteria

This narrative review summarized the available results and provided an overview of cinnamon’s effects on dysglycemia and dyslipidemia in type 2 diabetic subjects. Search was conducted from 2000 up to May 2022 in PubMed, Medline, and Cochrane Library databases, using the following keywords “cinnamon”, “glycemia”, “lipid”, “triglycerides”, “LDL”, “HDL”, “diabetes”, and “type 2 diabetes”. The framework window over two decades was considered taking into account the recent advances in clinical design. 

### 2.2. Data Charting Process

The article selection was employed by two independent studies through abstract review. The characteristic of studies (study design, study participants, interventions, and outcomes) was analyzed and reported. Besides glycemia and lipid parameters, other relevant biochemical parameters were considered. The variables analyzed in this study included: Fasting/postprandial blood glucose, hemoglobin A1c (HbA1c), anthropometric parameters (body mass index, body fat), and lipid parameters (total cholesterol, low-density lipoprotein cholesterol, high-density lipoprotein cholesterol, triglycerides). The screening process was divided into steps. Firstly, the articles were evaluated based on title and abstract review, and second, the articles were assessed based on full-text in order to verify if the inclusion criteria were included.

### 2.3. Eligibility Criteria

The articles that contain the following criteria were included in the present review: (i) Participants aged 18 years or older, (ii) subjects with T2DM diagnosis or impaired glycemia subjects and metabolic syndrome subjects, (iii) men or non-pregnant women participants, (iv) study design including experimental and/or quasi-experimental clinical trials, (v) studies with or without control group and (vi) cinnamon extract or powder. The exclusion criteria included no peer-reviewed articles, proceedings, and letters/comments. 

## 3. Results

### 3.1. Literature Search and Study Flowchart

**Figure 1:**
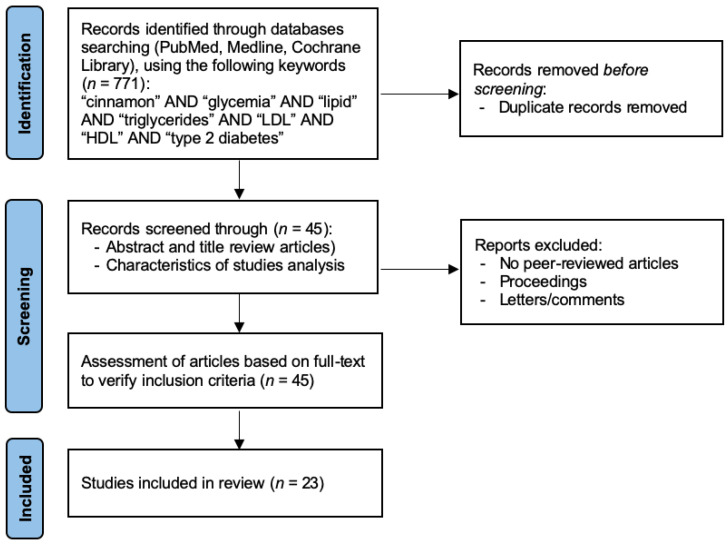
Flow diagram depicting the study selection process.

The search strategy initially identified a total of *n* = 771 articles from databases regarding the effect of cinnamon on glycemia and lipid profile. Following the title reviewed, *n* = 45 articles were selected: *n* = 26 and *n* = 19 articles for cinnamon effect on glycemia and lipid profile, respectively. Of those, after abstract analysis, a total of *n* = 14 full articles met the inclusion criteria for cinnamon effects on glycemia, and 9 full articles met the inclusion criteria for cinnamon effects on lipid profile. As a result, a total of *n* = 23 full articles were included in this narrative review (Figure 1). 

### 3.2. Cinnamon Effects on Glycemia

A total of 14 clinical trials on humans were analyzed in this review regarding the effect of cinnamon on blood glucose concentration in T2DM patients. Of those trials, eight studies demonstrated that cinnamon improved fasting blood glucose and postprandial blood glucose. The other six trials revealed that cinnamon administration had no beneficial effect on either fasting or postprandial blood glucose levels. It is important to note that only five studies employed a well-controlled clinical trial with a control group. It is noteworthy that this literature search included clinical studies of type 2 diabetic patients or impaired glycemia subjects.

In either diabetic or impaired fasting glycemia subjects, the ingestion of 1, 2, or 6 g of *C. cassia* after meals for 40 days by capsule administration decreased fasting glycemia by 18–29% at the end of the experimental protocol. However, the sulfonylurea drugs taken daily by the participants can also help in the improvement of fasting blood glucose levels observed in these participants [15]. A similar study reported that the ingestion of 6 g cinnamon in a capsule for 12 weeks did not significantly alter fasting blood glucose compared with the control group [16]. 

The administration of 2 g of *C. cassia* for 40 days also decreased PBG [17]. Furthermore, *C. zeylanicum* (3 g/day) and *C. cassia* (1 or 2 g/day) administration significantly decreased hemoglobin A1c (HbA1c) [18,19,20]. Aqueous cinnamon extract intake in different doses (250, 336, 360, and 500 mg) in capsule form for three or four months also resulted in fasting glycemia [21,22,23]. These results strongly support the significant blood glucose lowering beneficial effects of cinnamon in the treatment of T2DM. However, studies by Vanshoonbee et al. and Blavins et al. showed that cinnamon administration (1.5 g and 1 g, respectively) had no beneficial hypoglycemic effect [24,25]. In the study with impaired glucose tolerance subjects, the administration of 6 g cinnamon also demonstrated no significant effect on insulin response [26].

During an oral glucose tolerance test (OGTT), the ingestion of *Cinnamomun burmanni* aqueous extract did not significantly influence postprandial glycemia [27]. Table 1 summarizes the effects of cinnamon on glycemia and lipid profile in T2DM and impaired glycemia subjects. 

### 3.3. Cinnamon Effects on Lipid Profile

A total of nine clinical trials were analyzed regarding the effect of cinnamon on lipid profile in a human model. Most of these studies (six studies) revealed that cinnamon had no marked beneficial effect on diabetic subjects. However, in several animal studies, cinnamon was reported to lower blood lipids to significant levels.

Khan et al. demonstrated that the administration of either 1, 3, or 6 g for 40 days of *C. cassia* decreased triglycerides (TG) levels and total cholesterol (TC) levels [15]. Another study also suggests that the extract of *Cinnamomum aromaticum* (120 mg) by capsule significantly decreased TG levels at the end of three months compared at the beginning of the experimental protocol [22]. 

### 3.4. Cinnamon Effects on Inflammatory and Oxidative Parameters

Cinnamon seems to exert an important adjuvant effect on inflammation biomarkers and oxidative stress [28], which also contribute to the prevention of cardiovascular diseases (CVDs) and long-term complications in T2DM patients [29]. Cinnamon seems to possess a beneficial impact on systemic inflammation factors. The ingestion of cinnamon improved the plasma level of C-reactive protein (CRP), particularly in a chronic condition, which is characterized by elevated CRP levels [30]. Conversely to these results, cinnamon supplementation did not significantly improve CRP in diabetic patients [31]. Cinnamon Ceylon (600 mg) for 60 days significantly reduced interleukin-6 and nitric oxide [32] in the intervention group compared to the control group. In T2DM patients, a randomized, double blind, and controlled trial showed that 3 g cinnamon extract supplementation for eight weeks did not significantly reduce NF-KB, IL-6, and IL-8 and TNF-alpha [33].

Oxidative stress also has an important role in the development of T2DM-induced long-term complications, which can also contribute to beta cell destruction in t2DM patients [34]. After eight weeks of cinnamon administration, diabetic patients received no beneficial effects on total antioxidant capacity [31]. Conversely, the ingestion of 0.5 g of *Cinnamomum zeylanicum* combined with a probiotic significantly lowered advanced glycation end-products and the oxidized low-density lipoprotein (ox-LDL), compared with the control group. Moreover, cinnamon seems to improve antioxidant enzymes activity, especially catalase enzyme [35]. The administration of 250 mg of aqueous cinnamon extract for 12 weeks significantly increased antioxidant power by ferric reduction [21].

**Table 1:** A summary of the effects of cinnamon on glycemia and lipid profile of type 2 diabetic and impaired glycemia subjects.

References	Study-Design	Samples	Interventions	Antidiabetic Medications	Outcomes
Akilen et al. (2010) [20]	Randomized, double-blind, placebo trial	T2DM subjects (*n* = 58)	*Cinnamomum cassia* powder capsules (2 g per day), 4 times with meals, 12 weeks	Metformin, Sulphonylureas, Metformin and sulphonylureas	Comparison between intervention and control groups: ↓HbA1c (*p* < 0.005) No effect on lipid profile and FGL
Blevins et al. (2007) [25]	Randomized, placebo trial	T2DM subjects (*n*=43)	*Cinnamomum cassia* (1 g per day) capsule, 2 times with meal, 3 months	Sulfonylureas, meglitinides, metformin, thiazoledinediones, alpha-glucosidase inhibitors, exenatide, hydromethylglutaryl- CoA reductase inhibitors, ezetimibe, niacin, or fibric acid derivatives	No significant effect on FBG, lipid profile and HbA1c and insulin levels
Crawford et al. (2009) [19]	Randomized, placebo trial	T2DM subjects (*n* = 89)	*Cinnamomum cassia* capsule (1 g), 2 times with meal, 90 days	Oral antidiabetic agents, insulin	Comparison before and after intervention: ↓HbA1C (*p* < 0.001)
Khan et al. (2003) [15]	Randomized, placebo trial	T2DM subjects (*n* = 57)	*Cinnamomum cassia* (1, 3 or 6 g per day) capsule, after meals, 40 days	Sulphonylureas	Comparison before and after intervention: ↓FBL (*p* < 0.05) ↓TG (23–30%, <0.05), LDL (7–27%, <0.05), TC (12–26%, <0.05). No effect on HDL cholesterol
Lu et al. (2012) [22]	Randomized, double-blind, placebo trial	T2DM subjects (*n* = 66)	*Cinnamomum aromaticum* extract (120 mg and 360 mg per day) capsule, 3 months	Gliclazide	Comparison before and after intervention: ↓HbA1c (*p* < 0.01) and ↓FBG with both doses (*p* < 0.01) ↓TG (120mg) (*p* < 0.01). No effect on TC, HDL and LDL
Mang et al. (2006) [23]	Randomized, double-blind, placebo trial	T2DM subjects (*n* = 55)	Cinnamon aqueous extract (3 g/day) capsule, 3 times with meal, 4 months	Metformin, sulphonylureas, glinides, glitazones, combination therapies	Comparison before and after intervention: ↓FBG (*p* < 0.05) No effect on lipid profile and HbA1c levels
Roussel et al. (2009) [21]	Randomized, double-blind, placebo trial	Impaired fasting glycemia subjects (*n* = 22)	*Cinnamomum cassia* aqueous extract (250 mg/day) capsule, 2 times, 12 weeks	No diabetic therapeutic	Comparison before and after intervention: No significant effect on fasting insulin level ↓FBG (*p* < 0.05)
Sengsuk et al. (2016) [36]	Randomized, double-blind, placebo trial	T2DM subjects (*n* = 99)	Cinnamon powder placebo (500 mg per day), 3 times with meal, 60 days	Antidiabetic agents	Comparison between intervention and control groups: ↓ΔSystolic BP (*p* < 0.001) and ↓ΔDiastolic BP (*p* = 0.002) ↓ΔTG (*p* < 0.001), ↓ΔTC (*p* < 0.001), ↓ΔLDL (*p* = 0.178) ↓ΔHbA1c (*p* < 0.001)
Soni et al. (2009) [17]	Placebo-controlled trial	T2DM subjects (*n* = 30)	*Cinnamomum cassia* powder capsule (2 g per day), 4 times after meal, 40 days	Oral antidiabetic agents	Comparison before and after intervention: ↓FBG (*p* < 0.01 and ↓PBG (*p* < 0.01)
Talaei et al. (2017) [31]	Randomized, double-blind, placebo trial	T2DM subjects (*n* = 44)	Cinnamon powder capsule (1000 mg), 3 times with meal, 8 weeks	Metformin	Comparison between intervention and control groups: No effect in ΔFBG (*p* = 0.06) No effect in ΔHbA1c (*p* = 0.87)
Vafa et al. 2012) [18]	Randomized, double-blind, placebo trial	T2DM subjects (*n* = 44)	*Cinnamomum zeylanicum* (3 g per day) capsules, 3 times with meal, 8 weeks	Metformin; Gliclazide	Comparison before and after intervention: ↓HbA1c (*p* < 0.05), ↓FBG (*p* < 0.05) and TG (*p* < 0.05 Treated group vs. control: No significant effect in TC, HDL and LDL and anthropometric
Vanschoonbeek et al. (2006) [24]	Randomized, double-blind, placebo trial	T2DM subjects (*n* = 25)	*Cinnamomum cassia* (1.5 g per day) capsule, 3 times with meal, 6 weeks	Sulfonylureas derivatives, metformin, and/or thiazolidinediones	No significant effect in FBG, insulin levels, glucose level on OGTT, HbA1c and lipid profile (TC, HDL, LDL, TG)
Wickenberg et al. (2014) [16]	Randomized, double-blind, placebo trial	Impaired glucose tolerance subjects (*n* = 17)	*Cinnamomum cassia* (6 g per day), 2 times with meal, 12 weeks	No diabetic therapeutic	Comparison between intervention and control groups: No significant effect on insulin sensitivity, HbA1c, FBG
Zare et al. (2018) [37]	Randomized, triple-blind, placebo trial	T2DM subjects (*n* = 140)	Cinnamon powder (500 mg) 1 capsule, twice a day, 3 months	Oral hypoglycemic agents	Comparison between intervention and control groups: ↓ΔBMI (*p* < 0.001), ↓ΔBody fat (*p* < 0.001) ↓ΔFBG (*p* < 0.001), ↓ΔHbA1c (*p* < 0.001) ↓ΔTG (*p* = 0.05), ↓ΔTC (*p* < 0.001), ↓ΔLDL (*p* = 0.018)
Ziegenfuss et al. (2006) [38]	Randomized, double-blind, placebo trial	Pre-diabetes and metabolic syndrome subjects (*n* = 22)	Cinnamon aqueous extract (500 mg/day) capsule, 2 times with meal, 12 weeks	No diabetic therapeutic	Comparison before and after intervention: ↓FBG in cinnamon-treat group (*p* < 0.01)

T2DM—type 2 diabetes mellitus; BMI—body mass index; BP—blood pressure; PBG—postprandial blood glucose; FBG—fasting blood glucose; HbA1c—hemoglobin A1c; TC—total cholesterol; LDL—low-density lipoprotein, HDL—high-density lipoprotein; TG—triglycerides; OGTT—oral glucose tolerance test; ↑—increase; ↓—decrease.

## 4. Underlying Mechanism of Action of Cinnamon in T2DM

Several clinical trials have shown beneficial effects of cinnamon, suggesting its hypoglycemic and hypolipidemic properties [14,39]. For this reason, the main research on cinnamon has been focused on the prevention and treatment of T2DM as a diet complement [9,23]. While most studies have reported a beneficial metabolic effect of cinnamon on T2DM, its mechanisms of action are not yet fully established, and there are only isolated proposals, such as those taken from the literature and described below.

### 4.1. Glucose-Regulation Molecular Mechanisms of Cinnamon

Both *in vitro* and *in vivo* animal studies have reported marked beneficial effects of cinnamon and its bioactive compounds on glycemic control [40,41]. In several *in vivo* studies, cinnamon extract (200 mg/Kg bw) significantly decreased blood glucose levels in male C57BL/Ks db/db mice compared to C57BL/Ks db/db mice without cinnamon [42]. In addition, the administration of cinnamon aqueous extract (30 mg/Kg bw) significantly reduced glycemia in diabetic rats compared to diabetic rats without cinnamon [43]. One of the main bioactive compounds of cinnamon is cinnamaldehyde (20 mg/Kg bw), which has been shown to significantly improve HbA1c and plasma glucose levels in diabetic rats compared to diabetic rats without cinnamon [44]. These effects were also observed with the administration of cinnamon polyphenolic extract in T1DM [45]. Cinnamon oil can also decrease fasting blood glucose in T2DM rats [46]. In STZ-induced diabetic rats treated with polyphenol-rich de-coumarinated extract of cinnamon, there was a significant improvement in blood glucose levels and serum insulin compared to standard aqueous cinnamon extract containing 18% polyphenols content and 0.8% coumarin [47]. 

In light of the reported beneficial effects of cinnamon in the literature, it is now possible to postulate its potential mechanism(s) of action on glycemic control. The main mechanism of action of cinnamon is based on the hypothesis that it can elicit an insulin-mimetic-like effect through the regulation of insulin signaling pathways [48,49]. Thus, it is tempting to propose that cinnamon is exerting its beneficial effects on glucose homeostasis utilizing the following endogenous pathways:

(i) By increasing glucose uptake in muscle and adipose tissue by glucose transporter (GLUT) 4 production and GLUT 4 translocation [43,50].
(ii) By promoting glycogen synthesis in the liver, thereby inhibiting glycogen synthase kinase 3β [51] and
(iii) By decreasing gene expression of two regulators of gluconeogenesis in the liver, the phosphoenolpyruvate carboxykinase (PEPCK) and the glucose-6-phosphatase [52].

**Figure 2:**
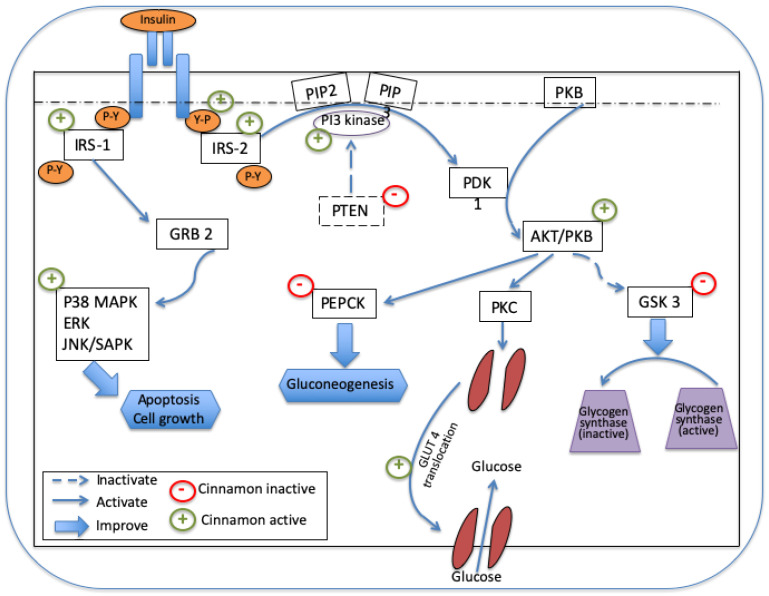
A schematic model illustrating the proposed mechanism(s) of action of cinnamon and its isolated bioactive compounds on glycemic control. Legend: IRS, insulin receptor substrate molecule; PIP2, phosphatidylinositol 4,5-biphosphate; PIP3, phosphatidylinositol 3,4,5-triphosphate; PI3 kinase, phosphatidylinositol 3-kinase; PTEN, phosphatase and tensin homolog; GRB2, growth factor receptor-bond protein 2; p38 MAPK, p38 mitogen-activated protein kinase; ERK, extracellular-signal-regulated kinases; JNK/SAPK, c-Jun *N*-terminal kinase/stress-activated protein kinase; PEPCK, phosphoenolpyruvate carboxy-kinase; PDK1, 3-Phosphoinositide-dependent kinase 1; PKB/AKT, protein kinase B; PKC, protein kinase C; GSK 3, glycogen synthase kinase 3; GLUT 4, glucose transporter protein.

The mechanism by which cinnamon and its bioactive components regulate insulin signaling includes the activation of intracellular cascade events. Thus, the extract of this plant and its isolated compounds (hydroxychalcone) seem to stimulate the insulin tyrosine kinase receptor (IR), tyrosine auto-phosphorylation [53], and then, the insulin receptor substrate molecules (IRS) [54]. The IRS-2 phosphorylation results in the activation of phosphatidylinositol 3-kinase (PI3K), which is responsible for the activation of phosphoinositide-dependent protein kinase (PDK1). This kinase, in turn, activates different signaling molecules, such as protein kinase B (Akt1/PKB), that has been reported to have an important role in the regulation of protein translocation, enzyme activity, and gene transcription of different enzymes [49]. The Akt1/PKB can enhance protein kinase C (PKC), which, in turn, stimulates glucose uptake by the cell. Similarly, Akt1/PKB inhibits GSK-3, leading to an activity of glycogen synthase. In addition, cinnamon also seems to inhibit the phosphatase and tensin homolog deleted on chromosome 10 (PTEN) that is responsible for inhibiting PI3K. Cinnamon extract can exert an ameliorated effect on GLUT 4 translocation via other signaling pathways in 3T3-L1 adipocytes. This includes the stimulation of the phosphorylation of AMP-activated protein kinase (AMPK) [55]. Finally, the effect of cinnamon on the IRS-1 insulin receptor substrate molecule can stimulate the P38-MAPK, ERK and JNK/SAPK signaling pathways via GRB-2, leading to apoptosis and cell growth [49]. All these multiple effects of cinnamon and its isolated compounds help in regulating the glycemia induced by T2DM. Figure 2 is a schematic model describing mechanism(s) of action of cinnamon and its isolated bioactive compounds on glycemic control.

*In vitro* studies have also suggested that cinnamon can exert an antidiabetic effect by gastro-intestinal enzyme inhibition, which contributes to reducing postprandial glycemia by a decrease in starch digestion. Cinnamon Ceylon significantly inhibited the alpha-amylase, and it was shown to be more efficient than other cinnamon species [56]. 

### 4.2. Lipid-Regulation Mechanisms of Cinnamon

Data from *in vitro* and *in vivo* animal studies have demonstrated that cinnamon can exert possible beneficial properties on lipid profile. In T2DM rats, aqueous cinnamon extract (200 mg/Kg) administration for six weeks can significantly decrease triglycerides (TG) and increase high-density lipoprotein (HDL) [42,57]. In addition, cinnamon oil administration can decrease total cholesterol (TC) after 35 days compared to the control group [46]. One of the bioactive compounds of cinnamon is cinnamaldehyde and it can also exert a beneficial effect on lowering the blood glucose levels in T2DM. In a dose of 20 mg/Kg, cinnamaldehyde can decrease TC levels, TG levels, and increase HDL [44]. 

**Figure 3:**
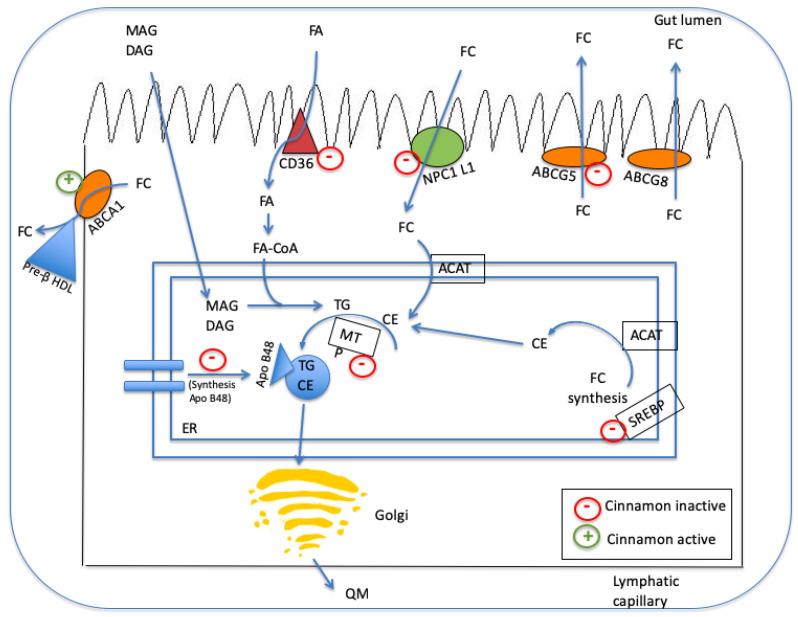
A schematic model describing mechanism(s) of action of cinnamon and its isolated bioactive compounds on lipid metabolism in the intestine. Legend: MAG, mono-acylglyceride; DAG, diacylglyceride; FA, fatty acid; FC, free cholesterol; NPC1 L1, Niemann–Pick C1-Like 1; ABCG, ATP-binding cassette sub-family G; ACAT, acetyl-CoA acetyltransferase; TG, triglycerides; CE, cholesterol ester; MTP, microsomal transfer protein; SREBP, sterol regulatory element binding protein; ER, endoplasmatic reticulum. QM, chylomicron.

The mechanism of action by which cinnamon and its compounds can regulate lipid profile metabolism is not clearly understood in the literature [58]. However, it is now possible to postulate its proposed mechanism(s) of action on lipid metabolism in the body. Figure 3 is a schematic model describing the possible mechanism(s) of action of cinnamon and its isolated bioactive compounds on lipid metabolism in the intestine.

A number of studies have identified some pathways involved in lipid metabolism employing enterocytes to study the effect of cinnamon and its isolated compounds [49]. Cinnamon can inactivate the Niemann–Pick c1-like 1 and Cd36 mRNA receptors on the enterocytes, leading to a decrease in the absorption of free cholesterol (FC) and free fatty acid (FFA), respectively, from the intestinal lumen to the enterocyte. Furthermore, cinnamon can exert a down-regulation of chylomicron synthesis by decreasing MTTP levels and Apo B48 secretion from the enterocytes, which are responsible for intestinal lipoprotein assembly. Cinnamon and its isolated compounds can also regulate cholesterol homeostasis by inducing ABCA1 expression, which, in turn, is responsible for promoting cholesterol efflux from enterocytes to the intestinal lumen. Cinnamon and its compounds can also decrease ABCG5 expression, which promotes cholesterol efflux from enterocytes into the intestinal lumen. Finally, cinnamon can down-regulate lipogenesis by decreasing Srebp 1c expression [49]. Together, all these multiple effects of cinnamon and its isolated compounds help in improving lipid profile and consequently in hyper-lipidaemia induced by T2DM.

## 5. Discussion

This review provides the available evidence from intervention trials evaluating the effect of cinnamon on dysglycemia and dyslipidemia in T2DM patients in the last two decades. Overall, the results from current literature search showed a considerable number of studies on the topic. The potential effect of cinnamon in T2DM-induced hyperglycemia has been extensively discussed. Cinnamon is an important additive to food consumed globally, and it has been widely reported to possess an anti-hyperglycemic and anti-hyperlipidemic properties. However, current scientific data suggest limiting and conflicting results in humans. The possible reason for the conflicting results may be due to the use of different doses, extracts, and species of cinnamon as well as different forms of administration (food or capsule) [59]. Furthermore, some other studies have analyzed the impact of cinnamon, and they reported that it could also be influenced by the variability of intervention time or the oral antidiabetic drugs used. 

Moreover, cinnamon has been employed as a potential therapeutic agent to treat T2DM, demonstrating its effectiveness in decreasing fasting blood glucose and insulin resistance improvement [59]. Although cinnamon has the ability to reduce fasting blood glucose levels, it nevertheless seems to alter the hemoglobin A1c (HbA1c) level with its supplementation. This result could be attributed to the short-term effects of cinnamon administration in different studies [59]. 

The published data on human studies have shown some discrepancies compared to animal studies since animal models have demonstrated marked beneficial effects on lipid profile. In this context, more studies should be performed, especially using well-controlled clinical trials with control groups for comparison.

In diabetic patients with dyslipidemia, the lipid profile could be modulated with cinnamon supplementation. Cinnamon supplementation seems to decrease serum triglycerides (TG), total cholesterol (TC), and low-density lipoprotein levels (LDL) [60]. These properties of cinnamon are assumed to play an essential role because dyslipidemia is the main risk factor for CVDs in diabetic patients [61].

Cinnamon extract also seems to have anti-antioxidant properties *in vitro*, showing an important capacity for oxidant protection [62]. However, few studies have been done in *in vivo* models evaluating the total serum antioxidant status. The results of this literature review in this study show that cinnamon seems to exert beneficial and protective anti-inflammatory and antioxidant effects. The possible bioactive compound responsible for these properties could be the proanthocyanins, a polyphenol compound [63]. This compound is presented in aqueous cinnamon extract and can exert a beneficial effect on the prevention of advanced glycation-end product (AGE) formation [64], which is originated by reactive oxygen species during the hyperglycemic status [33,65].

T2DM patients are treated mainly with anti-hyperglycemic drugs [66], combined with daily exercise, weight loss, and diet modification to obtain optimal metabolic outcomes [67]. These help in reducing blood glucose levels and lipid profile as well as preventing and delaying long-term complications due to T2DM. In this context, previous studies have suggested that cinnamon should be used as part of a dietary plan in conjunction with drug therapy for T2DM prevention and/or management [67]. Moreover, it is important to emphasize that cinnamon ingestion should be carefully determined so that it does not exceed the recommended doses per day [68].

Different mechanisms of action have been suggested in the scientific literature for cinnamon and its bioactive compounds regarding their beneficial properties. The main compounds identified in cinnamon are cinnamaldehyde, procyanidin type-A polymers, cinnamic acid, and coumarin. The polyphenol-rich cinnamon has been demonstrated to be a potential source of natural antioxidants, exhibiting strong free radical scavenging properties *in vitro*, which may contribute to protecting against oxidative stress [27].

Results from *in vitro* and *in vivo* models show that cinnamon can act beneficially as an antihyperglycemic agent through insulin-mimetic action via signaling pathway control [37,38]. Cinnamon seems to promote the GLUT4 translocation and glucose uptake in insulin-dependent tissue [43]. Furthermore, cinnamon can downregulate phosphoenolpyruvate carboxykinase (PEPCK) in the liver [43]. Another possible mechanism of action of cinnamon is related to a decrease in the intestinal enzyme activity affecting glucose absorption and thus the postprandial blood glucose concentration [41]. 

Moreover, cinnamon seems to be effective as an antihyperlipidemic agent. The mechanism of action proposed for this effect is related to the regulation of lipid metabolism in enterocyte. The bioactive compounds of cinnamon can also lower the absorption of cholesterol and fatty acid in gut cells through Niemann–Pick c1-like 1 and Cd36 mRNA receptors inactivation, respectively. Finally, cinnamon can also downregulate the chylomicron synthesis by decreasing microsomal triglyceride transfer protein (MTTP) levels and Apo B48 secretion from the enterocytes [49].

Further studies should be employed in order to understand the long-term effects of cinnamon and as part of daily meals in T2DM patients. Some of these include its ability to repair pancreatic beta cell mass, insulin synthesis and secretion, as well as its mimetic effects on L and K cells in the gut to activate the production and release of glucagon-like peptide-1 (GLP-1).

## 6. Concluding Remarks

Targeted cinnamon-based therapy can provide an opportunity to modulate glucose and lipid dysregulation in order to avoid the progression of T2DM. Cinnamon can also contribute as an antioxidant and an anti-inflammatory agent. However, there are controversial results in the scientific literature. Thus, future research studies should investigate the effect of cinnamon by employing a larger number of standardized randomized clinical trials in order to provide a comprehensive impact of cinnamon on diabetic patients. Additionally, a dose-response relationship should also be explored, taking in account that it is an important factor in disease prevention and/or treatment strategies.
